# Identification of the Porcine *G Protein-Coupled Receptor 41* and *43* Genes and Their Expression Pattern in Different Tissues and Development Stages

**DOI:** 10.1371/journal.pone.0097342

**Published:** 2014-05-19

**Authors:** Genlai Li, Hao Su, Zhenjin Zhou, Wen Yao

**Affiliations:** 1 Laboratory of Gastrointestinal Microbiology, College of Animal Science and Technology, Nanjing Agricultural University, Nanjing, Jiangsu, China; 2 Key Lab of Animal Physiology and Biochemistry, Ministry of Agriculture, Nanjing, Jiangsu, China; Inserm, France

## Abstract

Short-chain fatty acids (SCFAs) are not only an important energy source, but they also play a regulatory role in various physiological processes in humans and rodents. Current studies, mostly in humans and rodents, have revealed that SCFAs acted as endogenous ligands for *G protein-coupled receptor GPR41* and *GPR43*. Whether proteins similar to human *GPR41* and *GPR43* mediate the regulatory effects of SCFAs in swine remains unclear to date. The aims of this study were to determine whether *GPR41* and *GPR43* genes are expressed in porcine different tissues; and whether the expression of *GPR41* and *GPR43* is tissue-specific and/or time-associated. The alignment results showed that pig chromosome 6 contained GPR41 and GPR43 genes. Reverse transcription polymerase chain reaction (RT-PCR) indicated that GPR41 and GPR43 were expressed in porcine various tissues. The 2218 bp and 1908 bp nucleotide sequence representing the full-length *cDNA* sequence of porcine *GPR41* and *GPR43* was obtained from the ileum and spleen using rapid amplification of *cDNA* ends (RACE), which were capable of encoding 335 and 329 amino acid sequences, respectively. The structure prediction revealed that porcine *GPR41* and *GPR43* proteins had seven putative trans-membrane domains. The real-time PCR results indicated that *GPR41* and *GPR43* were expressed throughout the developmental stages in a tissue-specific and time-associated manner. *GPR41* and *GPR43* were most highly expressed in the ileum (*P*<0.01) and the spleen (*P*<0.01), respectively. Western blot results showed that porcine *GPR41* and *GPR43* proteins were expressed in a variety of porcine tissues, including the spleen, ileum, colon, and adipose tissue. In situ *GPR41* and *GPR43* immunoreactivities were observed through immunohistochemistry in the spleen, ileum, colon, and adipose tissue. In conclusion, the pig genome encoded *GPR41* and *GPR43* genes, and these two genes were detected in a variety of porcine tissues and expressed in tissue-specific and time-associated manner.

## Introduction

Short-chain fatty acids (SCFAs), predominantly acetate, propionate, and butyrate, can supply 15∼24% of net energy for maintenance in growing and finishing pigs [1], [2]. In addition to providing energy, SCFAs play a regulatory role in various physiological processes. Propionate is capable of inhibiting hepatic cholesterol synthesis in humans [3], reducing food intake [4], and improving tissue insulin sensitivity [5]. Propionate, along with acetate, may be involved in the regulation of adipogenesis [6] and increased cycling leptin level [7]. Butyrate enhances the differentiation and proliferation of colonic mucosa cells [8], ameliorates mucosal inflammation [9], and modulates visceral sensitivity [10].

The precise mechanisms underlying the above regulatory effects of SCFAs are poorly understood. Recent studies employing the “reverse pharmacology” approach have reported that SCFAs acted as endogenous ligands for orphan *G protein-coupled receptor 41* and *GPR43* [11]–[13]. It has also been reported that *GPR41* mediated the stimulatory effect of SCFAs on leptin production in adipocytes [7] and the effect of gut microbiota on host adiposity and energy balance in mice [14], and that *GPR43* mediated the effect of SCFAs on the promotion of adipogenesis [6] and inhibition of lipolysis in vitro [15]. Dewulf *et al*. [16] demonstrated that inulin-type fructans (ITFs), which can be fermented by gut microbiota for SCFA production, counteracted *GPR43* over-expression and *PPARγ* activation induced by a high-fat diet in the adipose tissue of mice. Bjursell *et al*. [17] found that *GPR43*-knockout protected mice from obesity and dyslipidemia induced by a high-fat diet. These rodents' original results indicated that *GPR41* and *GPR43* might be the underlying mechanism of SCFA-associated physiological processes.

To date, the majority of research studies on *GPR41* and *GPR43* have been on humans and rodents. The identification of porcine *GPR41* and *GPR43* and their functions in physiological processes remains to be elucidated. In this study, we tried to determine whether and where *GPR41* and *GPR43* genes are expressed in swine, and the expression pattern of these two genes in different tissues and developmental stages.

## Materials and Methods

All surgical and animal care procedures in this study followed protocols approved by Experimental Animal Care and Use guidelines (Chinese Science and Technology Committee, 1988).

### Tissue collection and RNA extraction

Various porcine tissues (liver, spleen, ileum, colon, heart, kidney, adipose tissue, and skeletal muscle) were collected from each three *Duroc×Landrace×Yorkshire* pigs slaughtered at one (newborn), 25 (weaning), 35 (nursing), 70 (nursing), 115 (growing), and 160 (finishing) days, and stored at −80°C until total RNA and membrane protein extraction.

### Reverse transcription polymerase chain reaction (RT-PCR)

The specific primers of *GPR41*, *GPR43*, and *GAPDH* genes (reference gene) were designed using Premier Primer 5 based on the sequences of predicted porcine *GPR41* (Accession number: XM_003127053.2) and *GPR43* (Accession number: XM_003127046.1), as well as *glyceraldehyde-3-phosphate dehydrogenase* (*GAPDH*, Accession number: NM_001206359.1). All primers used in this study were synthesized by Invitrogen (Shanghai, China) and are presented in Table 1.

**Table 1:** Primers sequences.

Primer	Sequences of primers
GPR41	TCTTCACCACCGTCTATCTCAC
	CACAAGTCCTGCCACCCTC
GPR43	CTGCCTGGGATCGTCTGTG
	CATACCCTCGGCCTTCTGG
GPR41-Q	GTTGGCATCCTGGCTGTT
	CCTCTTCTTCACCACCGTCTA
GPR43-Q	CGCTACCTGGGAGTGGCTT
	CGGCCTTCTGGGTTGAGTT
GAPDH	TTTGCGTCAGTGTCATCG
	TGCTCTGCCTTGGGTAAT
SP6	TAATACGACTCACTATAGG
T7	GATTTAGGTGACACTATAG
3' RACE-P1	GCAGTGGTATCAACGCAGAGTACTTTTTTTTTTTTTTTTTTTTTTTTTTVN
3' RACE-P2	GCAGTGGTATCAACGCAGAGTAC
3' RACE41-1	GCCAACGGGACCTGCTACCTG
3' RACE41-2	GATCAGCTGGCTCTTCTCCTGC
3' RACE43-1	CTGTCCCGCCGGCCCTTGTAC
3' RACE43-2	CTGTCGTGTTCATCGTTCAG
5' RACE-P1	GGCCACGCGTCGACTAGTACGGGGGGGGGGGGGG
5' RACE-P2	GGCCACGCGTCGACTAGTAC
5' RACE41-1	TGGGTAGGCCACGCTTAGGAA
5' RACE41-2	ACGGTGGTGAAGAAGAGGAAT
5' RACE43-1	CAAGGGCCGGCGGGACAGCT
5' RACE43-2	GCACGGGGAAAGCCACTC

RT-PCR was used to detect the expression of porcine *GPR41* and *GPR43* in various tissues. 30 µg of total RNA was pooled equally from three 160-day-old pigs and digested with 10 U of DNase I (Takara, Dalian) at 37°C for 30 min, followed by phenol-chloroform-isoamyl alcohol (25∶24∶1) and chloroform-isoamyl alcohol (24∶1) extraction. 2 µg of the DNase I-digested RNA were reverse-transcribed to *cDNA* in a total volume of 20 µL in the present or absence of PrimeScript RTase with oligo dT primer and random hexamers (Takara, Dalian). The conditions of these PCRs were 35 cycles of 98°C for 10 s, 60°C for 30 s, and 72°C for 30 s, followed by extension at 72°C for 10 min. The amplified products were detected by 2% agarose gel to characterize the distribution of *GPR41* and *GPR43* in porcine tissues.

### Rapid amplification of cDNA ends

Rapid amplification of *cDNA* ends (RACE) was used to amplify the 3′ and 5′ end regions of porcine *GPR41* and *GPR43* mRNA, using SMART RACE cDNA Amplification (Clontech, Beijing). The total ileum and spleen RNA from the three 160-day-old pigs was used for RACE of porcine *GPR41* and *GPR43*, respectively. For 3′ RACE, the first-strand *cDNA* was transcribed from 2 µg total RNA using the primer of 3′RACE-P1. The *cDNA* were used as the templates in subsequent nested PCR to amplify the 3′ end of porcine *GPR41* and *GPR43 cDNA* using parallel primer sets of 3′RACE P2 with 3′RACE 41-1 or 3′RACE 43-1, and 3′RACE P2 with 3′RACE 41-2 or 3′RACE 43-2 (Table 1). For 5′ RACE, 2 µg total RNA was pooled for the first-strand *cDNA* synthesis using the primer of oligo dT, and a TDT tail was added to the *cDNA*. The *cDNA* were used as the templates in subsequent nested PCR to amplify the 5′end sequences of porcine *GPR41* and *GPR43 cDNA* using parallel primer sets of 5′RACE P2 with 5′RACE 41-1 or 5′RACE 43-1, and 5′RACE P2 with 5′RACE 41-2 or 5′RACE 43-2 (Table 1).

### Plasmid construction and real-time PCR

The mRNA expression profiles of *GPR 41* and *GPR43* in the liver, spleen, ileum, colon, and adipose tissue with respect to the different developmental stages (1 d, 25 d, 35 d, 70 d, 115 d and 160 d) were determined with the real-time fluorescent quantitative PCR method, using an ABI PRISM 7300 Sequence Detection System (Applied Biosystems, NY).

The primers *GPR41*-Q and *GPR43*-Q were used to amplify porcine GPR41 and GPR43 genes from ileum and spleen *cDNA*, respectively. The amplified products were cloned to the pGEM-T Easy vector (Promega, Madison), which was subsequently transformed to TOP 10 competent cells (Tiangen, Shanghai). The plasmids with correct amplified products, as standard substances for absolute quantification of *GPR41* and *GPR43* mRNA, were extracted using a SunShineclean™ Plasmid Mini Extraction Kit (Sunshinebio, Nanjing). The calibration curves were performed using a series of diluted plasmid constructs. The slopes of the calibration curves were 3.53 and 3.25 for *GPR41* and *GPR43*, respectively, indicating that the efficiency of qPCR was acceptable (data not shown). There was only one amplified product for each pair of primers shown in melting curves, indicating that the primers we used were specific (data not shown).

One µg total RNA of various tissues from individual pigs at each development stage was first used for reverse transcription (PrimeScript RT reagent Kit with gDNA Eraser; Takara, Dalian). 2 µL of cDNA was mixed with 2× SYBR Premix Ex Taq with Tli RNaseH Plus (Takara, Dalian) using the primers *GPR41*-Q and *GPR43*-Q in a total volume of 20 µL. The conditions of these PCRs were 40 cycles of 95°C for 5 s and 58.5°C for 31 s, followed by a dissociation curve of 95°C for 15 s, 60°C for 1 min, 95°C for 15 s, and 60°C for 15 s. The qPCR data was analyzed with 7300 System SDS software v1.3.0 (Applied Biosystems, NY). The gene copies were calculated according to the calibration curves. The target gene expression results are presented as copies per 1 microgram total RNA [18].

### Western blot

About 100 mg of certain tissues were pooled equally from three 160-day-old pigs for membrane protein extraction using a Membrane and Cytosol Protein Extraction Kit (Beyotime, Nantong), according to the manufacturer's instructions, and protein concentration was determined using an Enhanced BCA Protein Assay Kit (Beyotime, Nantong). The extracted membrane protein was denatured with 5×SDS loading buffer at 95°C for 5 min and stored at −80°C until analysis. 40 µg denatured proteins were separated through a 12% SDS polyacrylamide gel and then transferred to a nitrocellulose membrane (Boster, Wuhan). After incubating in 5% nonfat dried milk for 2 h, the membrane was incubated with 1∶10000 HRP-conjugated monoclonal mouse anti-beta actin (Kangcheng, Shanghai) and 1∶200 polyclonal *GPR41* and *GPR43* antibodies (Santa Cruz Biotechnology, Texas) at 4°C overnight. The membrane was washed three times in TBST, and then incubated with 1∶10000 diluted horseradish peroxidase-conjugated anti-rabbit antibodies (Sunshinebio, Nanjing) for 1 h at room temperature. The membrane was again washed three times in TBST, after which it was incubated in Pierce Western blotting substrate (Pierce Biotechnology, IL) for 1 min. The chemiluminescent signals were visualized by Fujifilm LAS-4000 (Fujifilm, Tokyo).

### Immunohistochemistry

The distal ileum, colon, spleen, and adipose tissue were immersed in 4% paraformaldehyde. After fixation, the tissues were washed in 75% alcohol, dehydrated in a graded ethyl alcohol series (85%, 95% I, 95% II, 100% I, and 100% II), cleared in xylene, and embedded in paraffin. The tissues were serially sectioned into 4 µm-thicknesses on a rotary microtome. The paraffin sections were stained using an SABC kit (Boster, Wuhan, China), following the manufacturer′s instructions, and incubated with *GPR41* (1∶50 diluted) and *GPR43* (1∶50 diluted) antibody (Santa Cruz Biotechnology, Texas) at 4°C overnight. After immunoreaction, the images were captured on each slide at 400× magnification under a spot camera (Olympus, Tokyo). To check the specificity of the secondary antibody, the sections incubated without the primary antibody were stained by the secondary antibody as a negative control.

### Statistical analysis

The real-time PCR data were analyzed using JMP Pro 10. Multiple means were compared using Tukey's analysis. All the results are expressed as means ± standard deviation (SD). Differences were considered statistically significant at *P*<0.05, and extremely significant at *P*<0.01.

## Results

### Pig chromosome 6 contains *GPR41* and *GPR43* genes

A search of the pig genome database in GenBank (http://www.ncbi.nlm.nih.gov/genbank/) using the BLAST program (http://blast.ncbi.nlm.nih.gov/Blast.cgi) revealed that pig genome contains *GPR41* and *GPR43* genes, which are highly similar to these genes in humans, bovines, rats, and mice. The porcine *GPR41* and *GPR43* genes are located in tandem on chromosome 6. The similarities between porcine *GPR41* and human (NM_005304.3), bovine (NM_001145233.1), rat (NM_001108912.1), and mouse (NM_001033316.2) were 83%, 79%, 77%, and 88%, respectively. The similarities between porcine *GPR43* and human (NM_005306.2), bovine (NM_001163784.1), rat (NM_001005877.1), and mouse (NM_146187.3) were 85%, 83%, 81%, and 84%, respectively.

### Full-length amplification and tissue expression of porcine *GPR41* and *GPR43*

Full-length porcine *GPR41* was cloned from ileum cDNA, and *GPR43* was cloned from spleen cDNA by RACE, as the preliminary RT-PCR results showed that *GPR41* and *GPR43* were adequately expressed in the ileum and spleen, respectively. Nucleotide sequences of 2218 bp (Accession number: JX566878) and 1908 bp (Accession number: JX566880), representing the full-length cDNA sequences of porcine *GPR41* and *GPR43*, respectively, were obtained. The ORF finder (http://www.ncbi.nlm. nih.gov/gorf/orfig.cgi) was used to predict the open reading frame and the deduced amino acid sequence. The open reading frame of porcine GPR41 and GPR43 was 69∼1076 bp and 144∼1133 bp, respectively. Porcine *GPR41* mRNA was predicted to encode a 335-AA protein (Figure S1), while *GPR43* mRNA was predicted to encode a 329-AA protein (Figure S2). The multiple amino acid alignment results among human, bovine, rat, mice, and porcine *GPR41* and *GPR43* were processed using ClustalW2 multiple sequence alignment (http://www.ebi.ac.uk/Tools/msa/clustalw2/). The amino acid similarities between porcine *GPR41* and human, bovine, rat, and mouse *GPR41* were 71%, 82%, 73%, and 76%, respectively (Figure S3). The similarities between porcine and human, bovine, rat, and mouse *GPR43* were 82%, 80%, 83%, and 81%, respectively (Figure S4). The analysis results also indicated that these two proteins were membrane proteins containing seven trans-membrane domains.

**Figure 1:**
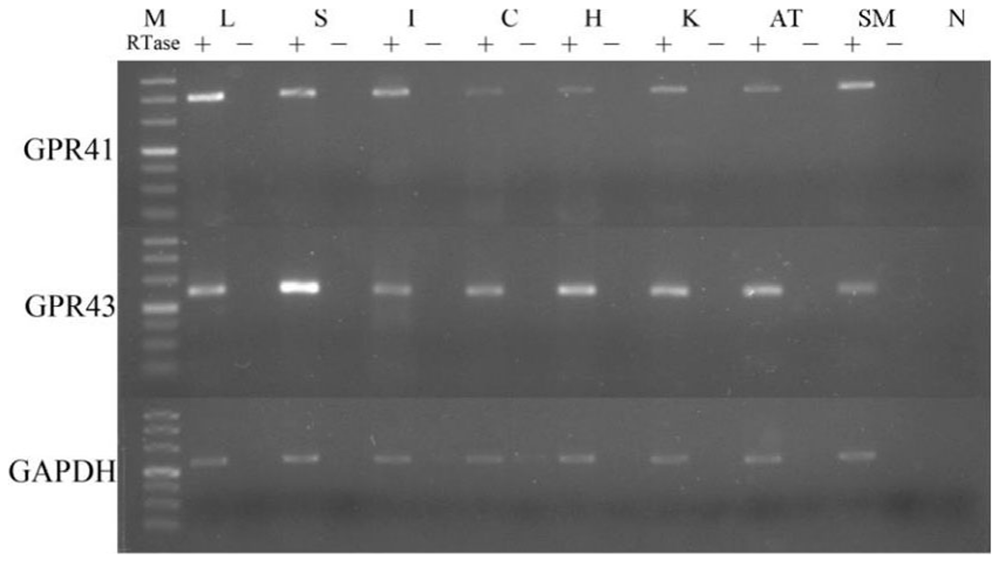
Expression of *GPR41* and *GPR43* mRNA in different porcine tissues at 160 day-old. The reverse transcription in the absence of reverse transcriptase (RTse –) was used to control for amplification from incompletely digested genome DNA. The *GAPDH mRNA* served as a loading control. The sizes of *GPR41*, *GPR43* and *GAPDH* PCR products were 398 bp, 249 bp and 220 bp, respectively. The bands of the marker were 50 bp, 100 bp, 150 bp, 200 bp, 300 bp, 400 bp, and 500 bp from bottom to above. M: marker; L: liver; S: spleen; I: ileum; C: colon; H: heart; K: kidney; AT: adipose tissue; SM: skeletal muscle.

The pooled RNA was used in this section. The pigs sampled in this study were of the same breed, from different families, of identical age and comparable body weight, and were raised in the same house using the same feed, so that the variation of gene expression would be negligible and using pooled RNA to describe the distribution of *GPR41* and *GPR43* in various tissues would be acceptable. The RT-PCR analysis indicated that both *GPR41* and *GPR43* mRNA were expressed in the tested tissues, including liver, spleen, ileum, colon, kidney, adipose tissue, heart, and skeletal muscle (Figure 1).

### Expression level of porcine *GPR41* and *GPR43* in different tissues and different developmental stages

**Figure 2:**
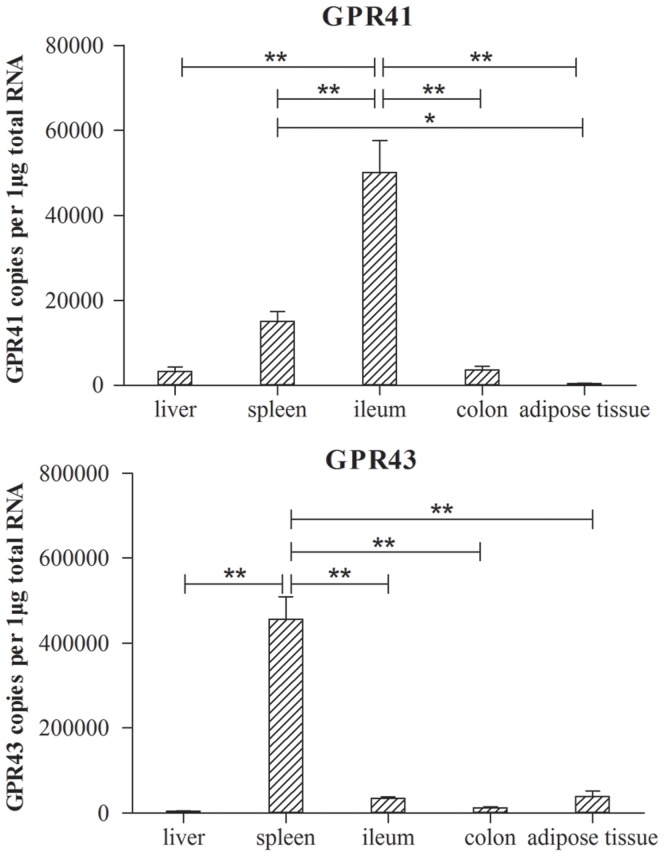
The expression level of *GPR41* and *GPR43* in different tissues (liver, spleen, ileum, colon and adipose tissue, n = 18) of pig. The vertical axis of the figure was presented as the target genes copies (*GPR41* and *GPR43*) per 1 microgram total RNA. Data was presented as mean ± SEM. Comparisons were made between different tissues (* *P*<0.05; ** *P*<0.01).

**Figure 3:**
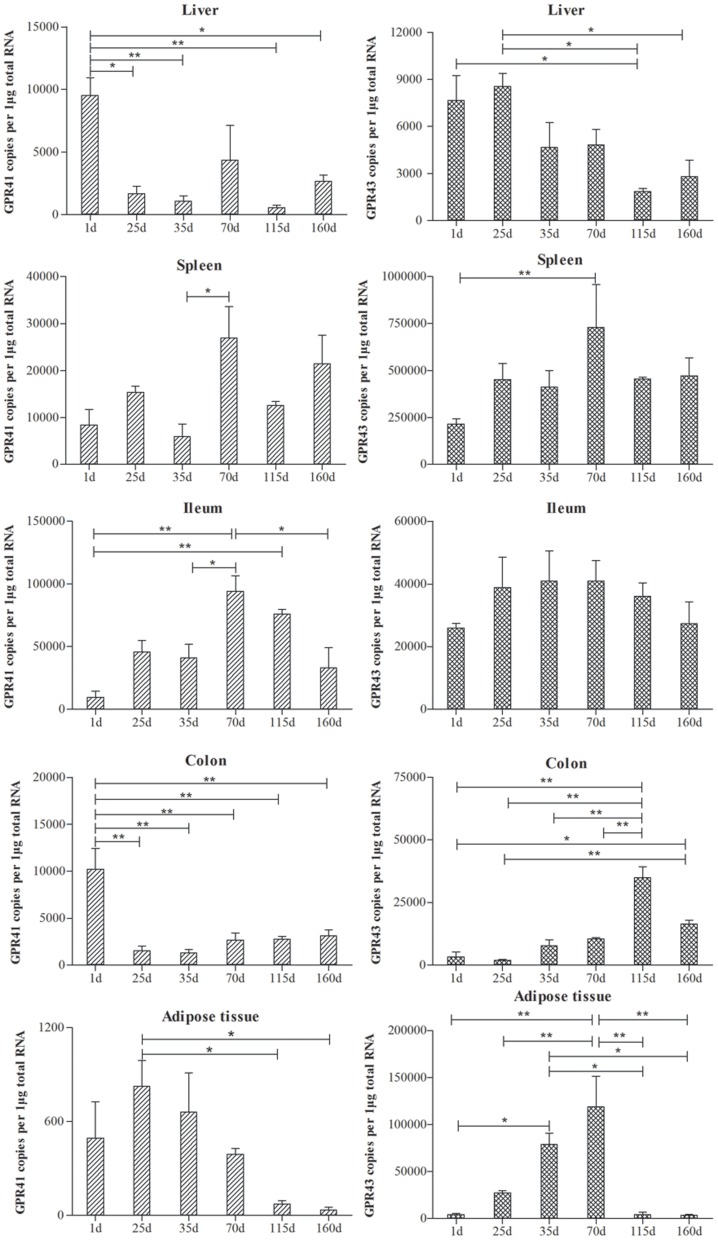
The expression level of *GPR41* (left) and *GPR43* (right) in liver, spleen, ileum, colon and adipose tissue at different development stages (0, 25, 35, 70, 115, 160 day) of pig (n = 3). The vertical axis of the figure was presented as the target genes copies (*GPR41* and *GPR43*) per 1 microgram total RNA. Data was presented as mean ± SEM. Comparisons were made between different developmental stages in each tissue (* *P*<0.05; ** *P*<0.01).

*GPR41* and *GPR43* were expressed in a significant tissue-specific and time-associated manner (Figures 2 and 3). *GPR41* was most adequately expressed in the ileum (Figure 2), which had a significantly higher expression level than any other tested tissue (*P*<0.01). Its expression level was higher in the spleen than in adipose tissue (*P*<0.05), and comparable in the liver, colon, and adipose tissue (*P*>0.05). The highest mRNA level of *GPR43* was in the spleen, which had a much higher expression level than the other tissues (*P*<0.01). There were no differences in *GPR43* expression level among the liver, ileum, colon, and adipose tissue (*P*>0.05).

Porcine *GPR41* and *GPR43* were also differentially expressed in different developmental stages (Figure 3). The highest expression levels of *GPR41* in the liver and colon were on the postnatal day, significantly higher than in the other developmental stages (*P*<0.05). After birth, the expression levels of *GPR41* in the liver and colon were down-regulated. The expression levels of *GPR41* in the spleen, ileum, and adipose tissue were up-regulated after birth, with peaks at 70 d in the spleen and ileum and 25 d in adipose tissue. The expression pattern of *GPR43* at different developmental stages was similar to that of *GPR41*. *GPR43* was highly expressed in the liver in the early developmental stages, whereas expression levels in the spleen, colon, and adipose tissue increased after birth, with peaks at 70 d, 115 d, and 70 d, respectively. However, *GPR43* expression in the ileum was comparable at the different developmental stages.

### Detection of porcine *GPR41* and *GPR43* protein by western blot and immunohistochemistry

**Figure 4:**
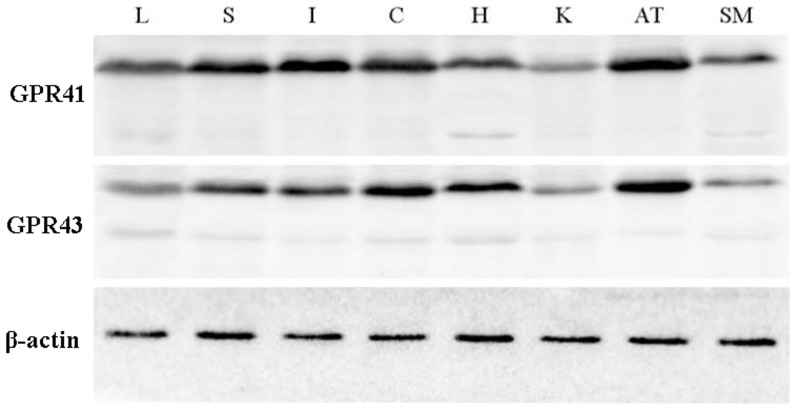
Analysis of *GPR41* and *GPR43* in porcine different tissues by western blot using *GPR41* and 43 antibodies. *β-actin* was used as the loading control. M: marker; L: liver; S: spleen; I: ileum; C: colon; H: heart; K: kidney; AT: adipose tissue; SM: skeletal muscle.

The tissue distribution of porcine *GPR41* and *GPR43* proteins was analyzed by western blot (Figure 4). The theoretical molecular weight of porcine *GPR41* and *GPR43* is about 40 kD; there were weak bands near 40 kD, but the predominant immunoreactive bands were located near 55 kD. Therefore, we considered that the porcine *GPR41* and *GPR43* proteins might be modified after translation, such as glycosylation and phosphorylation, which could result in a higher molecular weight than theoretical weight. NetPhos 2.0 Server (http://www.cbs.dtu.dk/services/NetPhos/) and NetOGlyc 3.1 Server (http://www.cbs.dtu.dk/services/NetOGlyc/) were utilized to analyze the phosphorylation and glycosylation sites of porcine *GPR41* and *GPR43*. The results showed that *GPR41* had 11 potential phosphorylation sites and one O-glycosylation site (Figure S1), while *GPR43* had nine potential phosphorylation sites and three O-glycosylation sites (Figure S2).

**Figure 5:**
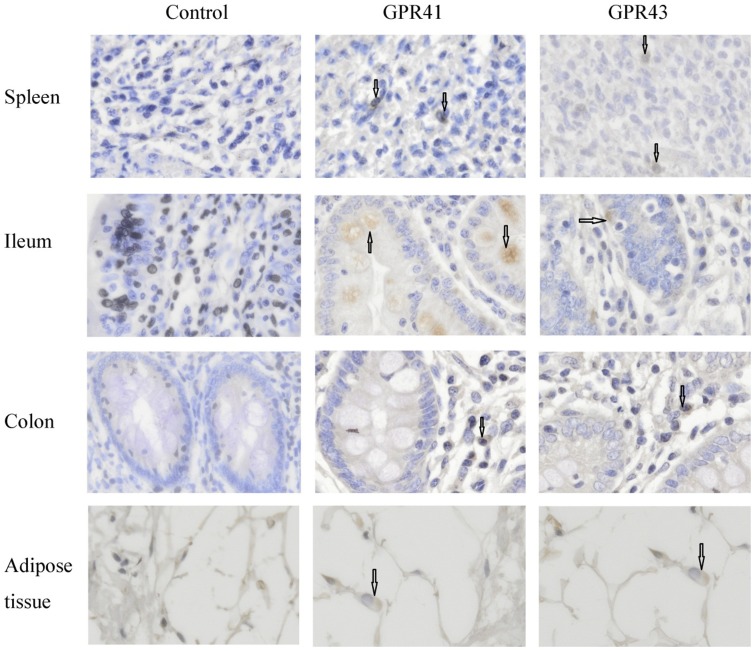
Immunolocalization of *GPR41* and *GPR43* in spleen, ileum, colon and adipose tissue. Sections were stained with the polyclonal human-specific *GPR41* and *GPR43* antibody (Santa Cruz, CA). The images were captured on each slide at 400× magnification under Olympus BX51 (Olympus, Japan). The cells marked by the arrows were *GPR41* or *GPR43* immunoactive. Bar = 20 µm.

To identify the cellular distribution of *GPR41* and *GPR43* in porcine tissues, immunohistochemical staining was performed using *GPR41* and *GPR43* antibodies. *GPR41*- and *GPR43*-immunoreactivities were observed in the spleen, ileum, colon, and adipose tissue (Figure 5), indicating that the cells in these tissues expressed *GPR41* and *GPR43* proteins.

## Discussion

As is known, SCFAs are generated by gut microbial fermentation of complex carbohydrates in the porcine distal small intestine and large intestine [19]. Acetate, propionate and butyrate are predominant SCFAs in the gut lumen of swine [19]. In addition to acting as the substrate for energy generation, SCFAs act as signal molecules and play a regulatory role in a variety of physiological processes. Given their critical regulatory roles, SCFAs and their regulatory functions have drawn much attention. However, the underlying mechanism of SCFAs remains unclear. The identification of SCFA receptors, *GPR41* and *GPR43*, might clarify the mechanism of SCFAs in various physiological processes.

*GPR41* and *GPR43* were firstly cloned by Sawzdargo *et al*. [20] in their search for the human galanin receptor subtypes. The *GPR40* family, including *GPR40*, *GPR41*, *GPR42*, and *GPR43*, was found to be located in tandem, downstream from *CD22* in chromosome 19q13.1 in humans [20]. Until 2003, three research groups had identified SCFAs as endogenous ligands for *GPR41* and *GPR43* [11]–[13]. Subsequent studies identified *GPR41* and *GPR43* in various tissues of several species, including humans [21], [22], rats [23], and bovines [24]. Our study focused on the identification of porcine *GPR41* and *GPR43* and their tissue distribution.

In the present study, a search of GenBank revealed that the pig genome contains *GPR41* (Accession number: XM_003127053.2) and *GPR43* (Accession number: XM_003127046.1) genes located in tandem in chromosome 6, and that they are highly similar to those of humans, bovines, rats, and mice. Some of the similarities among these genes were over 80%. However, most of the *GPR41* and *GPR43* mRNA sequences in GenBank are from a computational prediction, and the organization of *GPR41* and *GPR43* genes had not been carefully characterized in most of the species. The amino acid sequences of porcine *GPR41* and *GPR43* were deduced based on the sequences; these two nucleotide sequences encoded 335-AA and 329-AA protein, respectively. Multiple alignments showed that porcine *GPR41* and *GPR43* proteins are highly similar to those proteins in humans, bovines, rats, and mice. The structural prediction indicated that these two proteins were membrane proteins containing seven putative trans-membrane domains. However, the functions of porcine *GPR41* and *GPR43* are poorly understood, and subsequent research will focus on their functions in a variety of physiological processes.

RT-PCR results indicated that *GPR41* and *GPR43* were expressed in a variety of porcine tissues, including the spleen, ileum, colon, and adipose tissue. However, *GPR41* expression in adipose tissue is controversial. Le Poul *et al*. [12] detected *GPR4*1 expression in human adipose tissue, and Xiong *et al*. [7] observed that *GPR41* was expressed in mouse white adipose tissue and differentiated Ob-luc cells, but not in brown adipose tissue and undifferentiated Ob-luc cells. Conversely, Hong *et al*. [6] and Wang *et al*. [24] detected no expression of *GPR41* in adipose tissue, even though Hong *et al*. [6] used the same primers as Xiong *et al*. [7]. The reason for this discrepancy remains unclear; the inconsistent results might be due to differences in sample origins and techniques.

The RT-PCR results identified function genes on the mRNA level, but whether these genes play their role is determined by the translated proteins. Therefore, in this study, we characterized porcine *GPR41* and *GPR43* by western blot. The western blot analysis showed that the predominant bands for porcine *GPR41* and *GPR43* were higher than the theoretical molecular weights, suggesting that these two proteins might be modified after translation. The porcine *GPR41* and *GPR43* amino acid sequences had several potential phosphorylation and glycosylation sites, which were analyzed by online software. The results indicated that porcine *GPR41* and *GPR43* proteins might be modified after translation and result in target bands with higher molecular than theoretical weights as indicated by western blotting. Tazoe *et al*. [22] also detected human *GPR41* protein near 53 kD by western blotting. When PNGase was used to treat human *GPR41* protein, the protein shifted to a lower molecular weight, about 50 kD, indicating that it was indeed glycosylated [22]. However, 50 kD was also higher than the theoretical molecular weight, indicating that other unknown modifications might exist.

In our *in situ* immunohistochemical study, *GPR41* and *GPR43* immunoreactivity cells were detected in all tested tissues (spleen, ileum, colon, and adipose tissue). These results were partly in line with published results in humans and rats, in which *GPR41* and *GPR43* were co-localized with *5-HT-* and *PYY-*containing enteroendocrine cells in the ileum and the colon [21]–[23]. However, we were unable to confirm the type of *GPR41*- and *GPR 43*-immunoreactivity cells by the present staining.

Our quantitative real-time PCR results revealed the highest expression levels of porcine *GPR41* in the ileum and of *GPR43* in the spleen. Tazoe *et al*. [22] reported that *GPR41* was localized in human colon epithelial cells and *PYY*-containing enteroendocrine cells. *PYY*, which mediates SCFAs in the inhibition of upper gastrointestinal motility, is released by L cells in the mucosa of the gastrointestinal tract, especially in the ileum and the colon [25]. *PYY* is also known to be an important appetite control hormone, inhibiting food intake by means of a satiety signal [26]. In this study, porcine *GPR41* was most highly expressed in the ileum, suggesting that it might mediate the effects of SCFAs on *PYY* secretion and *PYY-*regulated functions. However, whether porcine *GPR41* is co-localized with *PYY*-containing enteroendocrine cells was not directly proved by our immunohistochemistry assay; further research is needed to clarify this question. Porcine *GPR43* was adequately expressed in the spleen, suggesting that *GPR43* might be implicated in host immune function. Brown *et al*. [27] also reported the highest expression levels of *GPR43* in the spleen in humans and rodents. Maslowski *et al*. [28] investigated the functions of *GPR43* in host immune response and proposed that the SCFA–*GPR43* interactions might represent a central mechanism to account for the effects of diet and gut microflora on immune responses and that they may represent new avenues for understanding and potentially manipulating immune responses.

The qPCR results also showed that the expression levels of *GPR41* and *GPR43* varied at different developmental stages of the tested tissues. The development of porcine gut microbiota and the SCFA-producing capacity at different ages might cause this time-associated manner of expression. The concentration of SCFAs in the gut lumen is quite low at birth, due to the low density of gut microbiota. Afterwards, the gastrointestinal tract is colonized by a variety of bacteria, including *Lactobacilli*, *Streptococci*, and *Enterobacteria* [29]. The abundant diversity and high population of gut microbiota induces a great deal of SCFA generation, which may up-regulate their receptors' expression. However, in most of the tested tissues, the expression levels of *GPR41* and *GPR43* were down-regulated in the growing and finishing phases. This finding might indicate that porcine *GPR41* and *GPR43* mainly exert their functions in the early developmental stages, especially before 70 days. Hong *et al*. [6] proved that *GPR43* is directly involved in adipocyte differentiation in vitro by observing the up-regulated expression levels of *GPR43* mRNA in differentiated adipocytes, with a peak at seven days, accompanied by an increase in *PPAR*γ*2* and leptin expression. The growth and development of adipose tissue include adipocyte differentiation and hypertrophy. Between 1 and 2 months of age, the increase in adipose tissue was primarily due to the introduction of new adipose cells, while between 5 and 6.5 months, the increase in adipose tissue was mainly due to the increase in cell size [30]. Our results showed that the levels of *GPR41* and *GPR43* mRNA were higher between 25 d and 70 d, indicating that porcine *GPR41* and *GPR43* might play a critical role in adipocyte differentiation. However, Hou *et al*. [31] observed that the expression level of *GPR43* in adipose tissue of Guangzhong black pigs up-regulated with age, and that the expression level at 10 months was significantly higher than at 2 and 5 months. The difference in the breed of experimental animals (Guangzhong black pig is a fatty-type Chinese pig with a strong capability for depositing fat) might account for the discrepancy. Further studies are necessary to clarify their function in porcine lipid metabolism.

In summary, our study has shown that the pig genome encodes *GPR41* and *GPR43* genes, and that these two genes are expressed in a variety of porcine tissues, including the spleen, ileum, colon, and adipose tissue. The expression of *GPR41* and *GPR43* occurs in a significant tissue-specific and time-associated manner, suggesting that these two receptors may have different functions in different tissues and at different developmental stages. The SCFA–*GPR41* and –*GPR43* interactions might also represent a novel link between gut microbiota and physiological processes. However, further research is required to determine the precise mechanisms of action of *GPR41* and *GPR43* in various physiological pathways.

## Supporting Information

Figure S1 Nucleotide and deduced amino acid sequences of porcine *GPR41*. The full-length of porcine *GPR41* was a 2218 bp nucleotide sequence (Accession number: JX566878), amplified from ileum *cDNA*, which encoded a 335-AA protein. The protein coding region was 69∼1076 bp. Porcine *GPR41* protein had seven putative trans-membrane protein and these seven trans-membrane domains were shadowed in this figure. The amino acids labeled with square icons are the potential phosphorylation sites, and labeled with circle icons are the potential Glycosylation sites.
(TIF)

Figure S2 Nucleotide and deduced amino acid sequences of porcine *GPR43*. The full-length of porcine *GPR43* was a 1908 bp nucleotide sequence (Accession number: JX566880), amplified from spleen *cDNA*, which encoded a 329-AA protein. The protein coding region was 144∼1133 bp. Porcine *GPR43* protein had seven putative trans-membrane protein and these seven trans-membrane domains were shadowed in this figure. The amino acids labeled with square icons are the potential phosphorylation sites, and labeled with circle icons are the potential Glycosylation sites.
(TIF)

Figure S3 Multiple alignments of porcine *GPR41* amino acid sequences (Accession number: AFV50552.1) with other known *GPR41* (human, bovine, rat and mouse). The GenBank accession number for the protein of human, bovine, rat and mouse are AAI13696.1, DAA19942.1, NP_001102382.1 and AAI25010.1, respectively.
(TIF)

Figure S4 Multiple alignments of porcine *GPR43* amino acid sequences (Accession number: AFV50553.1) with other known *GPR43* (human, bovine, rat and mouse). The GenBank accession number for protein of human, bovine, rat and mouse are AAH96200.1, DAA19940.1, NP_001005877.1 and AAH19570.1, respectively.
(TIF)
