# Correction: Identification of the Porcine *G Protein-Coupled Receptor 41* and *43* Genes and Their Expression Pattern in Different Tissues and Development Stages

**DOI:** 10.1371/journal.pone.0239768

**Published:** 2020-09-22

**Authors:** Genlai Li, Hao Su, Zhenjin Zhou, Wen Yao

After publication of this article [1], concerns were raised that in Fig 5, the adipose tissue panels for *GPR41* and *GPR43* appear to be derived from the same image.

**Fig 5:**
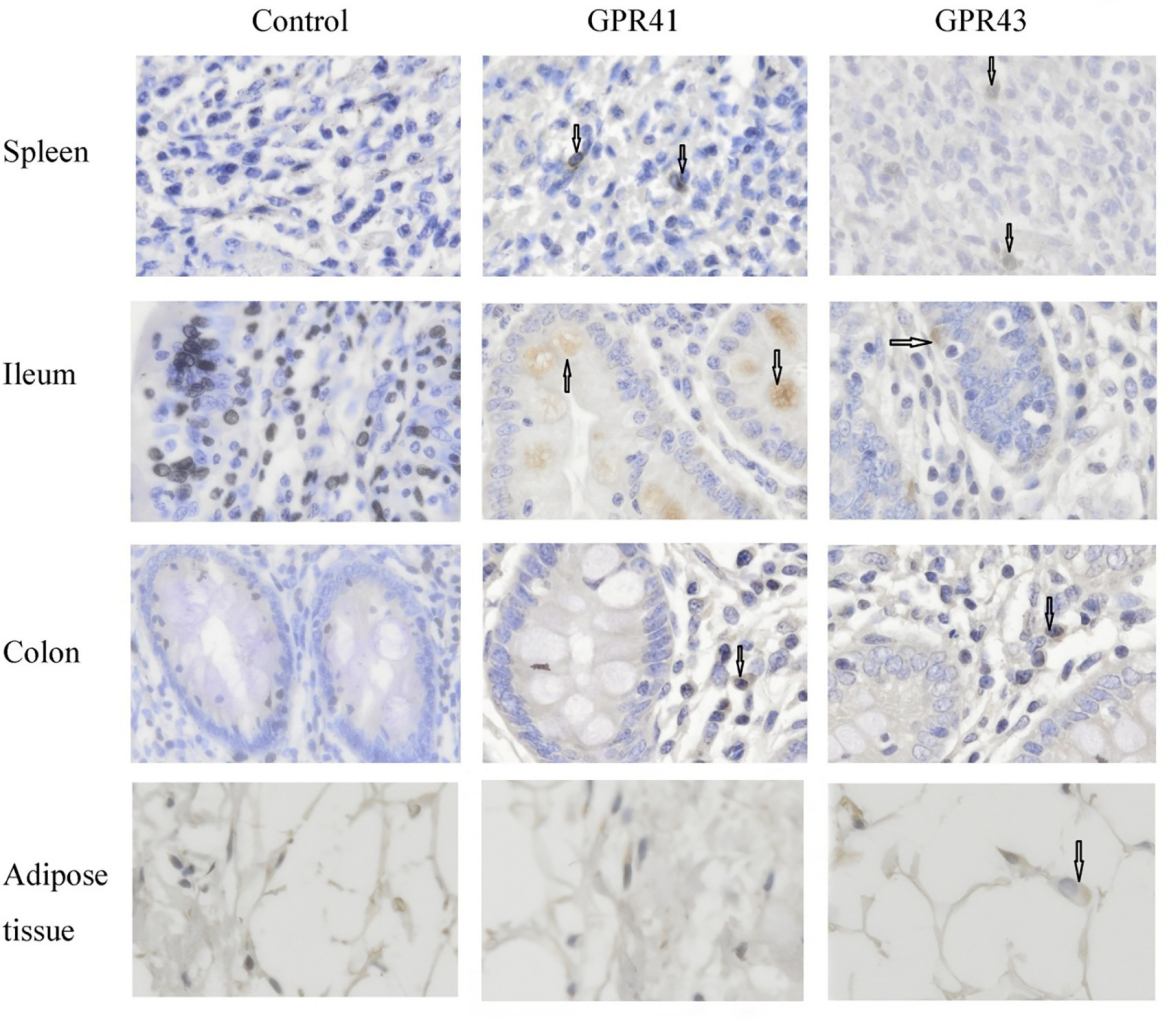
Immunolocalization of *GPR41* and *GPR43* in spleen, ileum, colon and adipose tissue. Sections were stained with the polyclonal human-specific *GPR41* and *GPR43* antibody (Santa Cruz, CA). The images were captured on each slide at 400x magnification under Olympus BX51 (Olympus, Japan). The cells marked by the arrows were *GPR41* or *GPR43* immunoactive. Bar = 20 μm.

The authors apologize that an error was made in generating Fig 5 such that the wrong image file was included for *GPR41* adipose tissue. The original images for *GPR41* and *GPR43* adipose tissue are provided in S1 and S2 Files, and the authors provide here an updated version of Fig 5 including the correct data. The original images supporting other panels of Fig 5 are no longer available.

An Academic Editor reviewed the updated figure and underlying data and confirmed that they support the results and conclusions reported in the published article.

The authors apologize for the error in the published article.

With this Correction, the authors also provide underlying data supporting Fig 2 and Fig 3 of [1], in S3 and S4 Files.

## Supporting information

S1 File Original image for *GPR41* adipose tissue results shown in the corrected version of Fig 5. (TIF)

S2 File Original image supporting *GPR43* adipose tissue results shown in Fig 5. (TIF)

S3 File Raw quantitative data supporting Fig 2 and Fig 3. (PZF)

S4 File Raw quantitative data supporting Fig 3. (PZF)
