# Supplementary figures and images for: Identification of the Porcine G Protein-Coupled Receptor 41 and 43 Genes and Their Expression Pattern in Different Tissues and Development Stages
Source: PLoS One. 2014 May 19;9(5):e97342. doi: 10.1371/journal.pone.0097342 (PMC4026140; doi:10.1371/journal.pone.0097342)

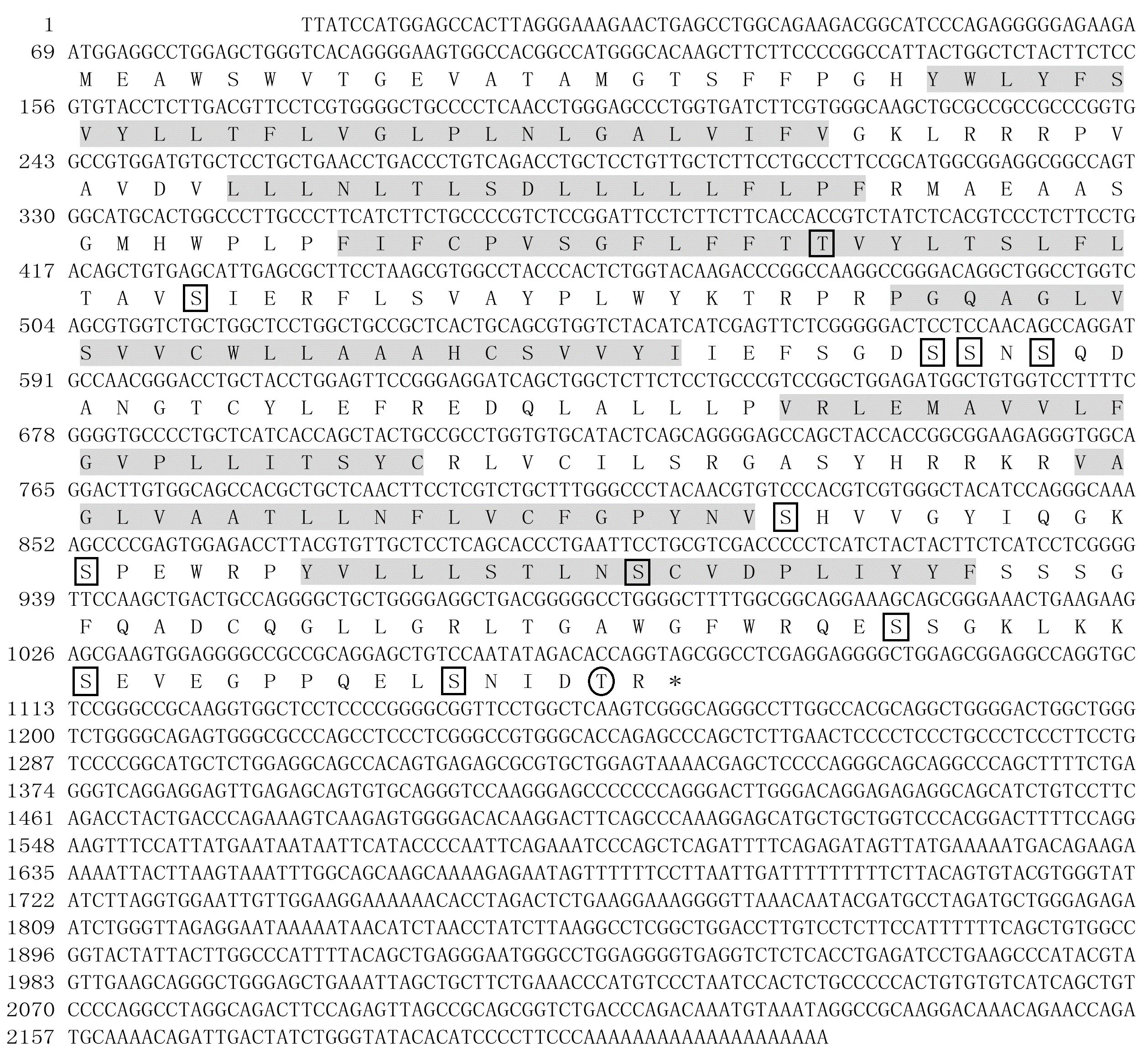

Supplement: Figure S1 — Nucleotide and deduced amino acid sequences of porcine GPR41. The full-length of porcine GPR41 was a 2218 bp nucleotide sequence (Accession number: JX566878), amplified from ileum cDNA, which encoded a 335-AA protein. The protein coding region was 69∼1076 bp. Porcine GPR41 protein had seven putative trans-membrane protein and these seven trans-membrane domains were shadowed in this figure. The amino acids labeled with square icons are the potential phosphorylation sites, and labeled with circle icons are the potential Glycosylation sites. (TIF) [file pone.0097342.s001.tif]

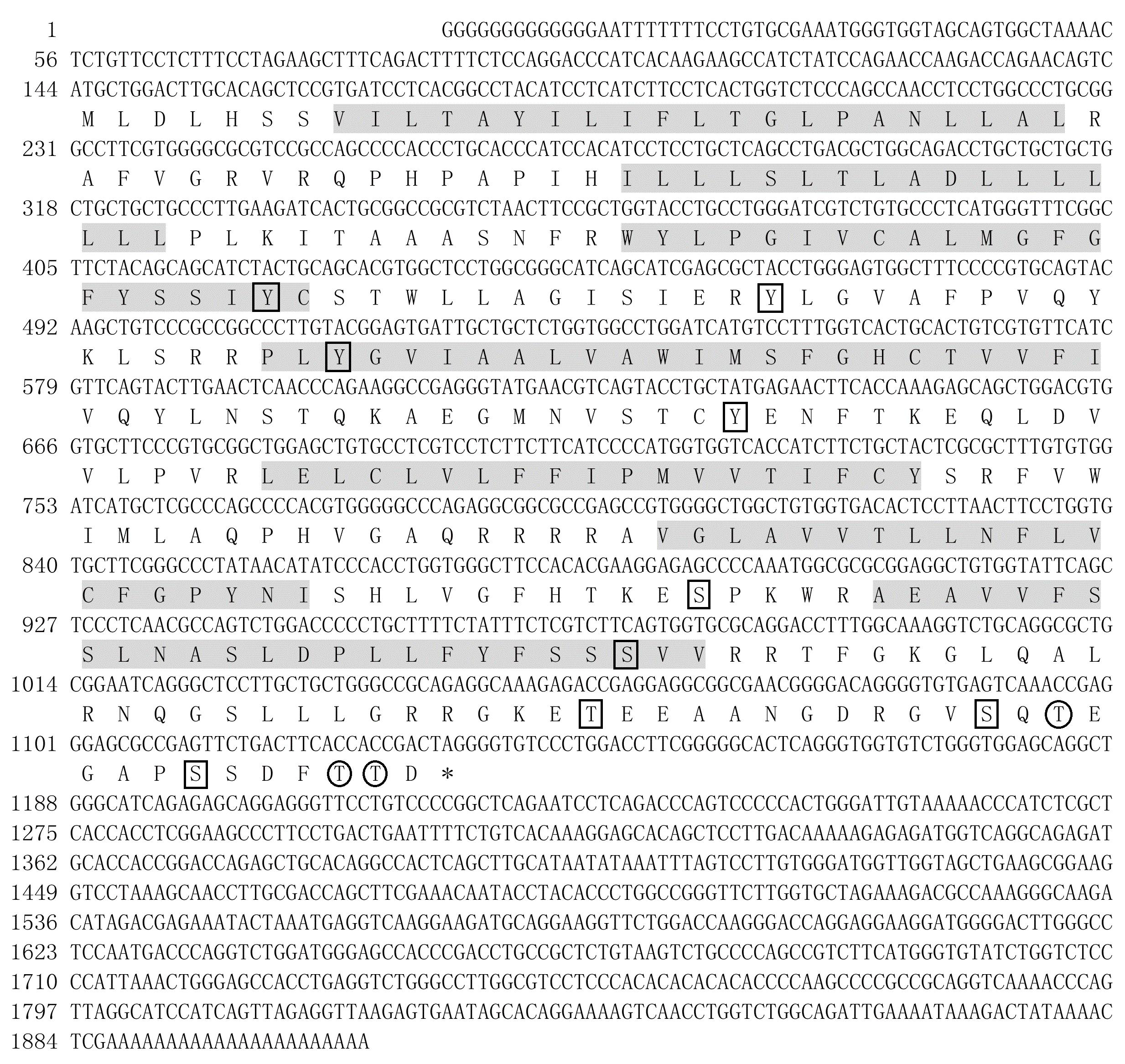

Supplement: Figure S2 — Nucleotide and deduced amino acid sequences of porcine GPR43. The full-length of porcine GPR43 was a 1908 bp nucleotide sequence (Accession number: JX566880), amplified from spleen cDNA, which encoded a 329-AA protein. The protein coding region was 144∼1133 bp. Porcine GPR43 protein had seven putative trans-membrane protein and these seven trans-membrane domains were shadowed in this figure. The amino acids labeled with square icons are the potential phosphorylation sites, and labeled with circle icons are the potential Glycosylation sites. (TIF) [file pone.0097342.s002.tif]

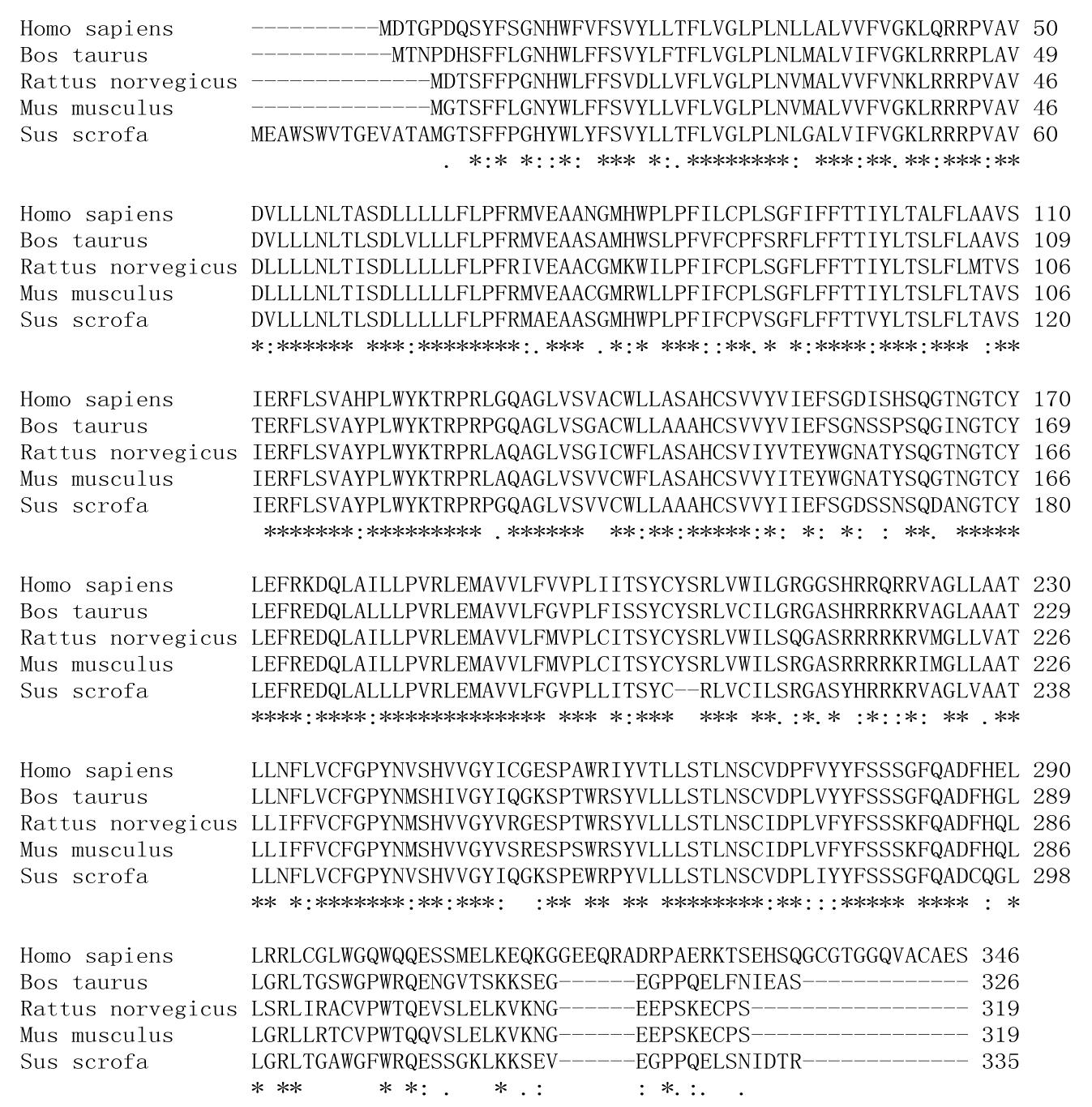

Supplement: Figure S3 — Multiple alignments of porcine GPR41 amino acid sequences (Accession number: AFV50552.1) with other known GPR41 (human, bovine, rat and mouse). The GenBank accession number for the protein of human, bovine, rat and mouse are AAI13696.1, DAA19942.1, NP_001102382.1 and AAI25010.1, respectively. (TIF) [file pone.0097342.s003.tif]

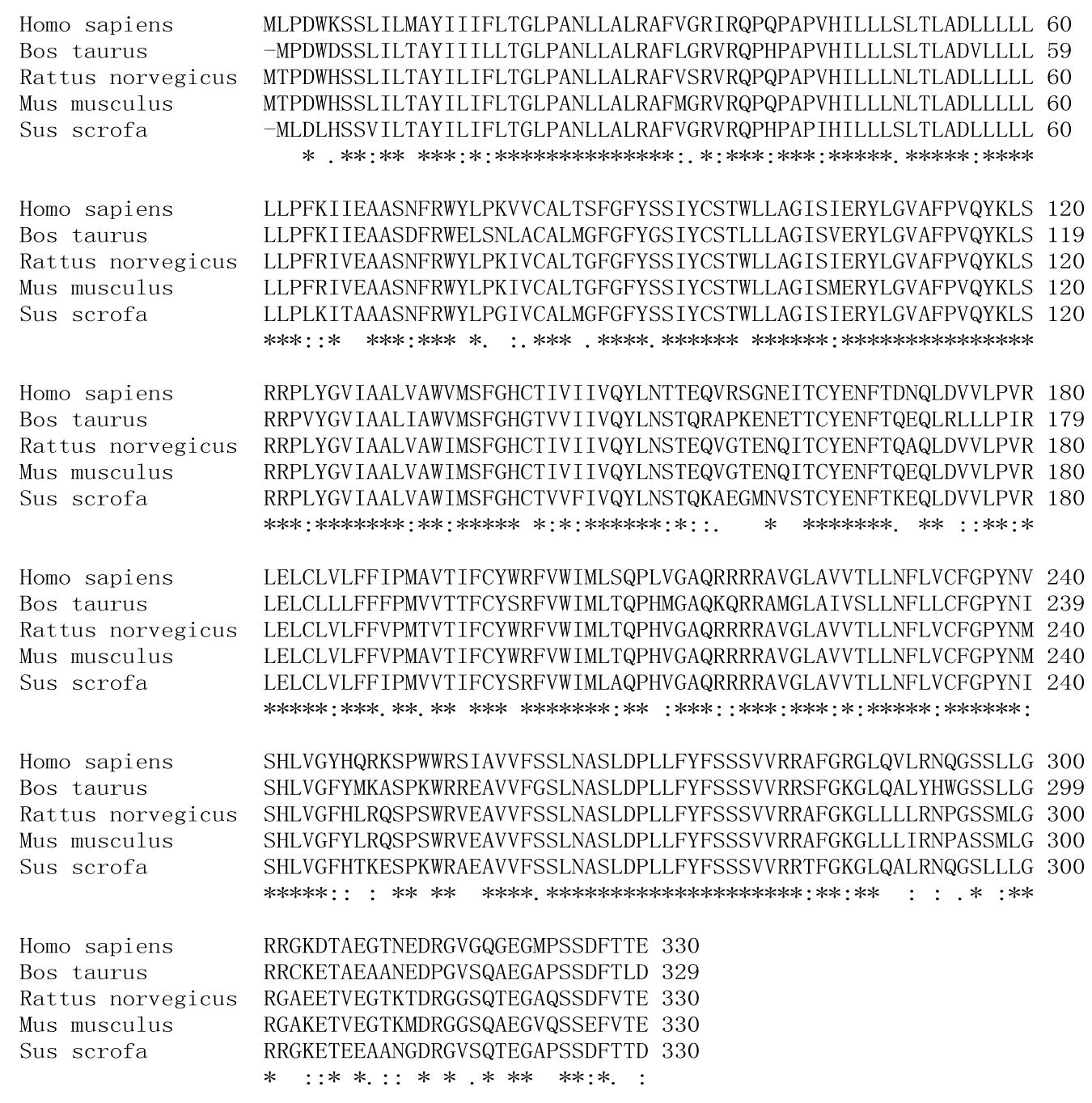

Supplement: Figure S4 — Multiple alignments of porcine GPR43 amino acid sequences (Accession number: AFV50553.1) with other known GPR43 (human, bovine, rat and mouse). The GenBank accession number for protein of human, bovine, rat and mouse are AAH96200.1, DAA19940.1, NP_001005877.1 and AAH19570.1, respectively. (TIF) [file pone.0097342.s004.tif]
