# Supplementary figures and images for: Correction: Identification of the Porcine G Protein-Coupled Receptor 41 and 43 Genes and Their Expression Pattern in Different Tissues and Development Stages
Source: PLoS One. 2020 Sep 22;15(9):e0239768. doi: 10.1371/journal.pone.0239768 (PMC7508376; doi:10.1371/journal.pone.0239768)

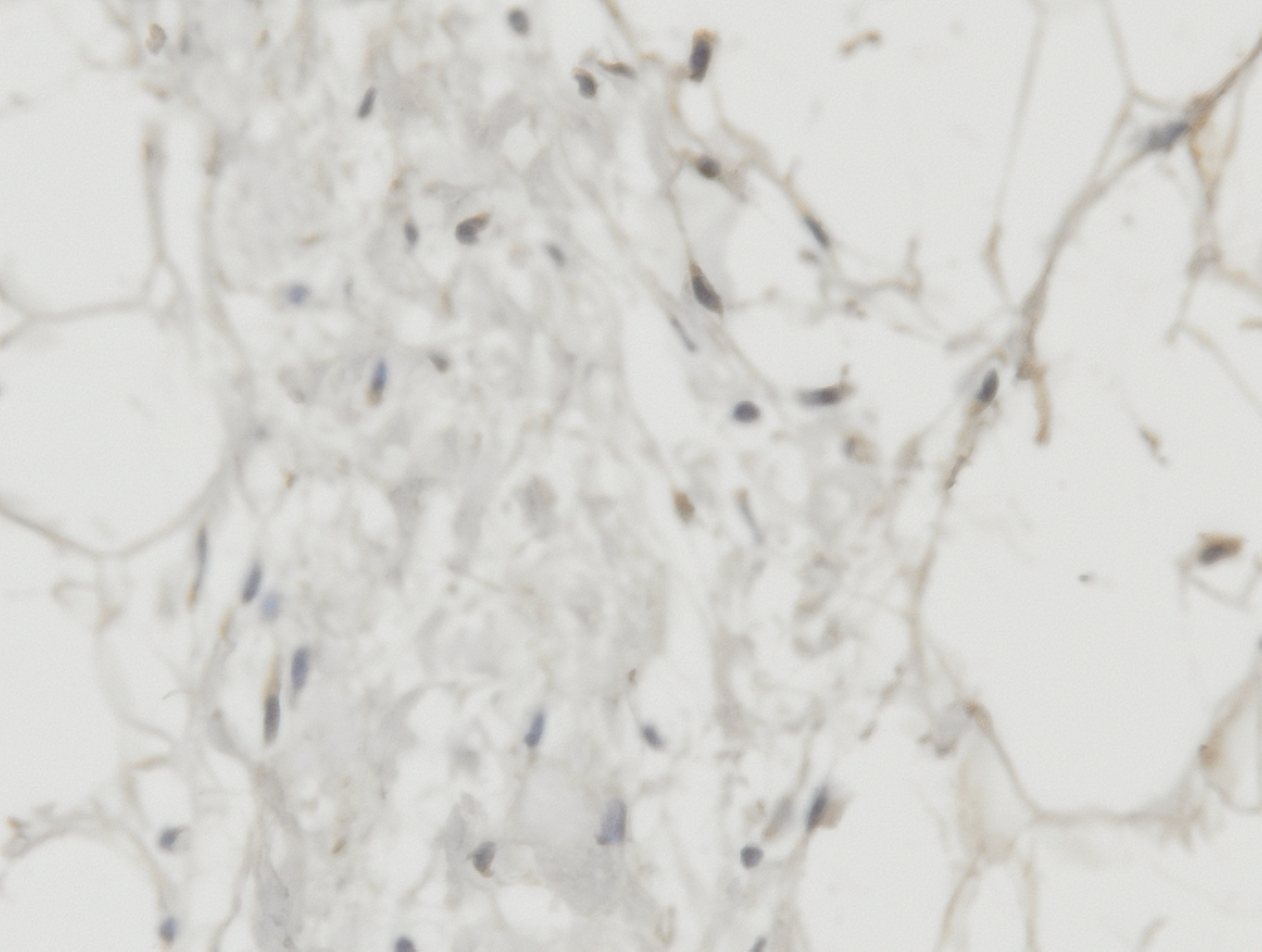

Supplement: S1 File — (TIF) [file pone.0239768.s001.tif]

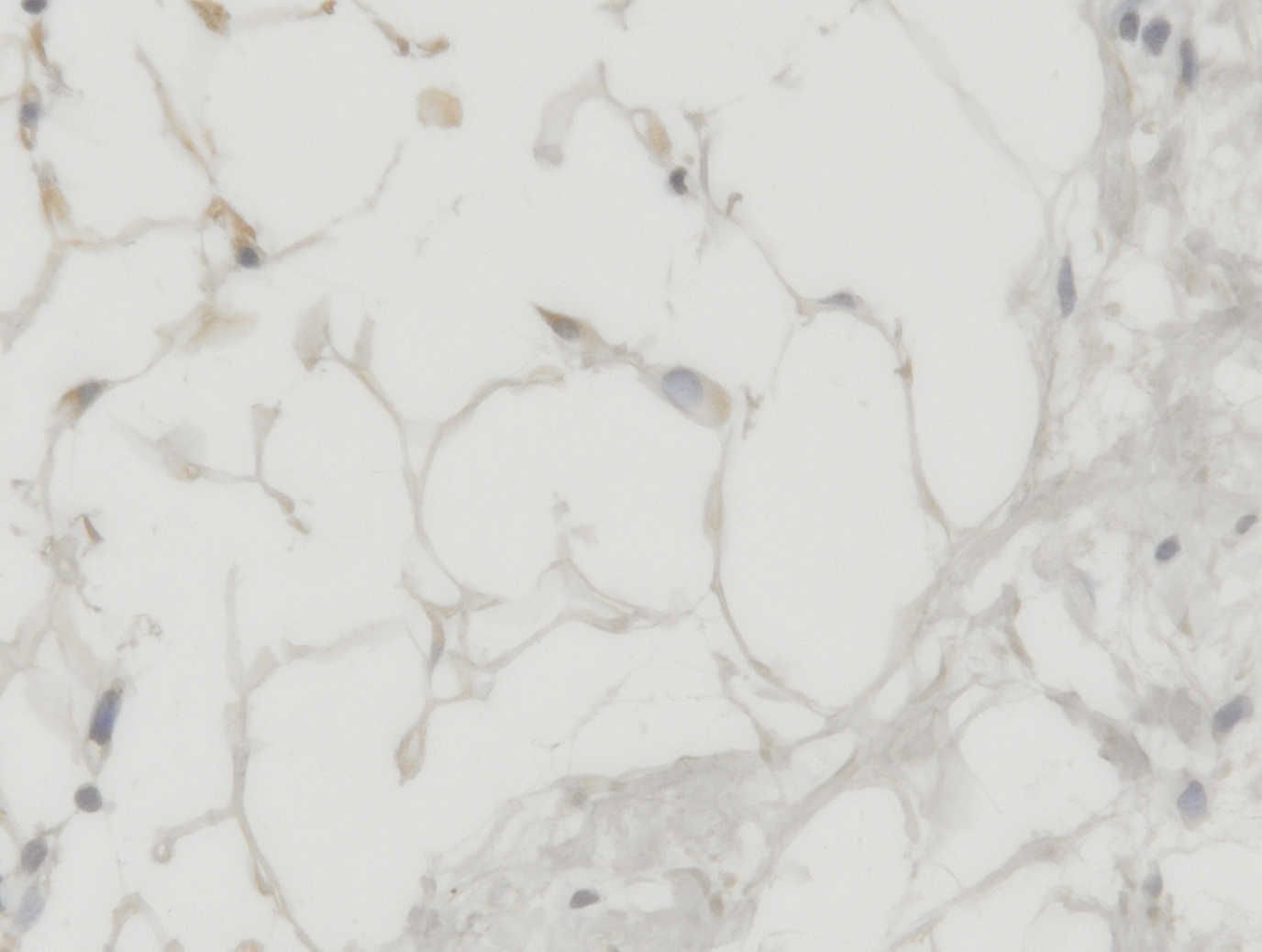

Supplement: S2 File — (TIF) [file pone.0239768.s002.tif]
